# Two Cases of Singular Sacral S1 Butterfly Vertebra

**DOI:** 10.3390/diagnostics15212775

**Published:** 2025-10-31

**Authors:** Arturs Balodis, Roberts Tumelkans, Cenk Eraslan

**Affiliations:** 1 Institute of Diagnostic Radiology, Pauls Stradins Clinical University Hospital, LV-1002 Riga, Latvia; 2 Department of Radiology, Riga Stradins University, LV-1007 Riga, Latvia; 3 Faculty of Medicine, Riga Stradins University, LV-1007 Riga, Latvia; 4 Radiology Department, Ege University, 35040 Bornova, Turkey

**Keywords:** sacral butterfly vertebra, vertebral anomaly, magnetic resonance imaging

## Abstract

A butterfly vertebra is an uncommon but clinically and radiologically significant pathology. The etiological factor of this pathology is a congenital defect in the formation of the vertebral body during embryogenesis, resulting in a cleft within the vertebral body that, in an X-ray, resembles the shape of a butterfly. Butterfly vertebrae are most often found in the thoracic and lumbar spine and more rarely in the sacral region. The clinical manifestations of this condition do not differ from the symptoms of other diseases, and it may also be asymptomatic. Only the recognition of its characteristic radiologic signs allows for accurate and timely diagnosis, as well as differentiation from other pathological processes such as fractures, metastases, and inflammation. In these cases, magnetic resonance imaging is the first-choice method. An important aspect in recognizing this pathology is its correlation with other congenital syndromes, even in cases of a single vertebral defect. We present 2 cases with an isolated S1 butterfly vertebra. The first is a 47-year-old male who presented to the hospital with complaints of chronic pain in the lower back and sacral region, more pronounced on the right side. The second is of a 39-year-old male who also presented to the hospital with chronic pain. All diagnostic modalities for this pathology have been used to demonstrate high-quality pictures, including X-ray, computed tomography (CT), and magnetic resonance imaging (MRI).

**Figure 1:**
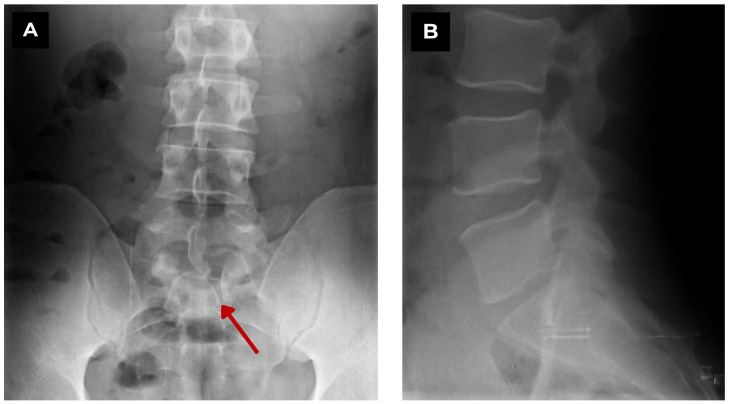
First patient. Lumbar spine X-ray of a 47-year-old male. (**A**) Coronal view. A vertebral defect can be seen at the left paramedian S1 level. (**B**) Sagittal view. No vertebral pathology can be seen. **Red arrow**—S1 butterfly vertebra. When performing an X-ray, two planes are necessary, as diagnosis can be very challenging. We aim to present clear, illustrative diagnostic images, covering all diagnostic modalities that fit the “interesting images” format, including high-quality X-ray, CT, and MRI, including full spinal MRI. Studies show that in the majority of patients (61%), only a single butterfly vertebra is observed, while in 39% of cases, multiple vertebral butterfly anomalies are present [1]. In this case, only a single butterfly S1 vertebra was diagnosed. In the literature, S1 butterfly vertebra anomalies are described in approximately 6% of cases [1]. It is reported that in around 50% of cases of this pathology, other congenital syndromes are observed, such as anterior meningocele, spina bifida, Alagille syndrome, Jarcho–Levin syndrome, Crouzon syndrome, and others [1,2]. Studies demonstrate a strong correlation between the presence of multiple butterfly vertebrae and other congenital syndromes; however, even in cases with a single butterfly vertebra, this association is observed in about one-third of patients [1,2,3,4]. In this presented case, no such correlation with other syndromes was observed.

**Figure 2:**
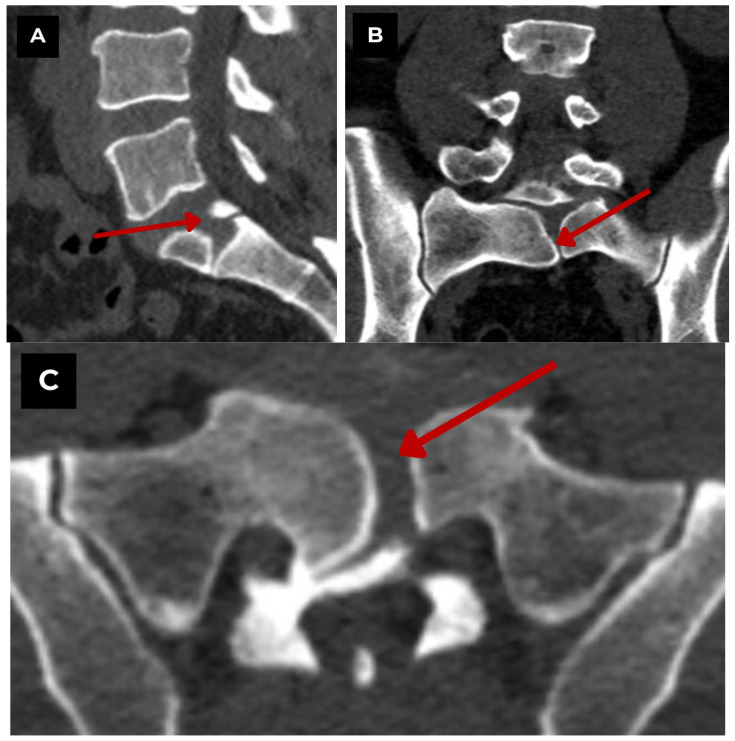
First patient. Lumbar and sacral CT bone window of a 47-year-old male. (**A**) CT sagittal. Shows a midline sagittal cleft extending through the S1 vertebral body. (**B**) CT coronal. Symmetric lateral hemivertebrae separated by a vertical cleft. (**C**) CT axial. Characteristic butterfly configuration. Findings suggest a benign, congenital vertebral anomaly with no evidence of acute fracture or destructive process. **Red arrow**—S1 butterfly vertebra.

**Figure 3:**
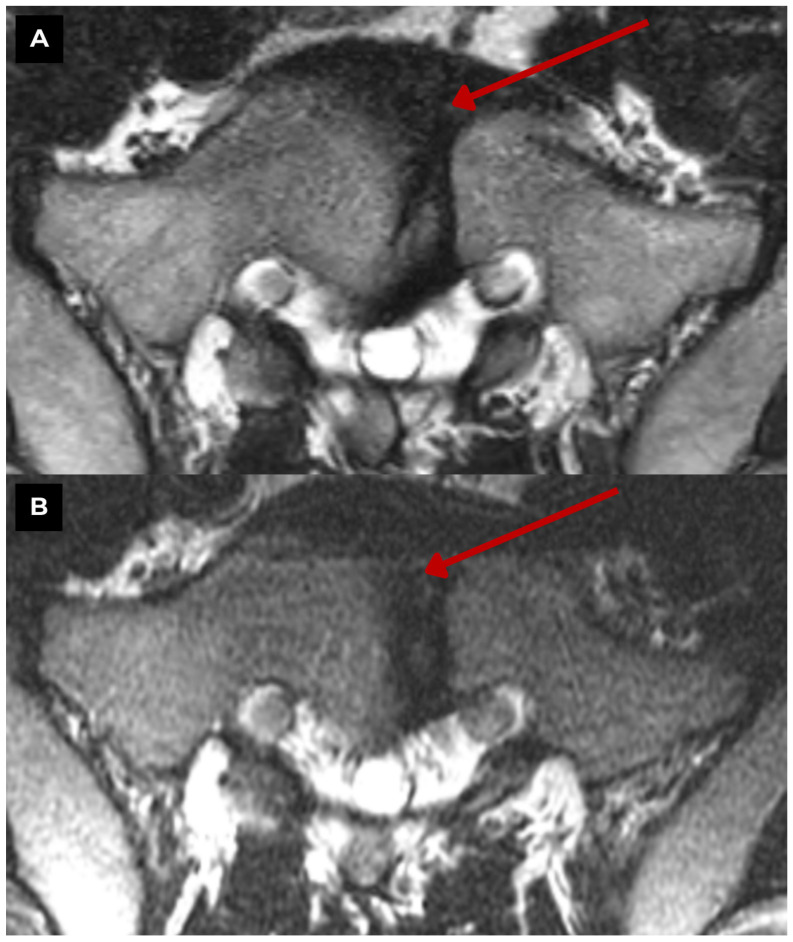
First patient. S1 vertebra MRI of a 47-year-old male. (**A**) T2-weighted sequence axial zoomed in. 4.8 mm paramedian defect slightly left of the midline. The S1 vertebra is altered, with a defect in its central part (central cleft), to be assessed as a butterfly vertebra, already visible in a previous MRI showing no changes over a one-year period in (**B**) T2-weighted sequence axial. There is no evidence of other congenital vertebral anomalies at any other level. **Red arrow**—S1 butterfly vertebra. The appearance of a butterfly vertebra can be variable. It is characterized by a wedge-shaped form and may cause local kyphosis [1]. A sagittal cleft is observed within the vertebral body, with funnel-shaped vertebral margins and characteristic symmetrical changes, although asymmetry of the margins may also be present [1,2].

**Figure 4:**
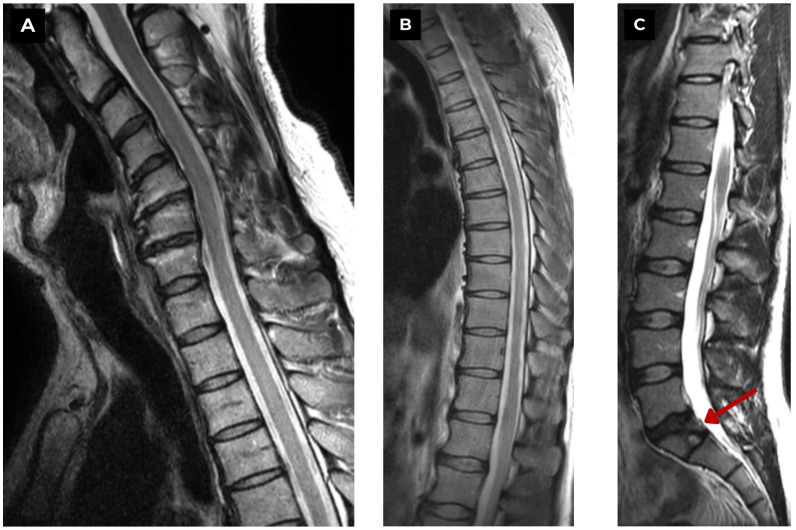
First patient. Full spinal MRI of a 47-year-old male. (**A**) T2-weighted sequence and (**B**) T2-weighted sequence. Show no vertebral anomalies in the cervical and thoracic regions; however, pronounced spondylosis is observed in the C3-C6 cervical vertebrae with a kyphotic deformity and slight uncovertebral hypertrophy. (**C**) T2-weighted sequence. Shows an S1 vertebra pathology with a central defect—butterfly vertebra. No acute degenerative changes or edema were seen in the short tau inversion recovery (STIR) sequence. **Red arrow**—butterfly vertebra. In T1WI, T2WI, and STIR MRI sequences (T1WI and STIR not shown for this patient), characteristic radiological signs of the vertebral cleft and intervertebral disc material are observed, as indicated by an isointense signal on T1WI and low signal intensity on T2WI and STIR [2,3]. The absence of signal intensity changes in the soft tissue and vertebrae on MRI helps differentiate this congenital pathology from other conditions such as inflammation, tumors, and trauma [5,6].

**Figure 5:**
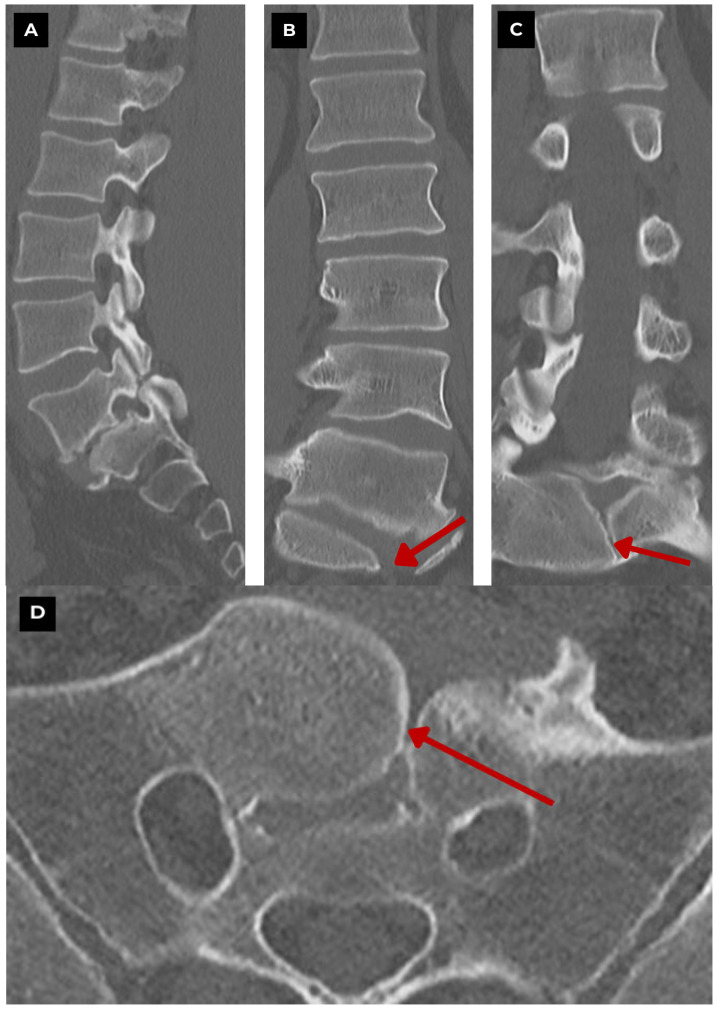
Second patient. Lumbar and sacral bone window CT of a 39-year-old male. (**A**) CT sagittal. (**B**,**C**) CT coronal. Show a cleft extending through the S1 vertebra body with sclerotic margins. Associated findings include mild scoliosis, anterolisthesis, and unilateral spondylolysis of the L5 vertebra. (**D**) CT axial. Shows a midline cleft consistent with a butterfly vertebra. Similarly to the first patient, there were no other congenital vertebral anomalies found elsewhere. **Red arrow**—butterfly vertebra. Although this is a case of an isolated vertebral anomaly, the altered spinal geometry might disrupt spinal biomechanics, potentially leading to curvature changes, slippage, and stress on the vertebra that might predispose to spondylolysis. Such associations are documented in the literature, with scoliosis being mentioned as the most frequent deformity found [1,7,8], while altered loading and alignment may contribute to chronic pain and disc slippage [9].

**Figure 6:**
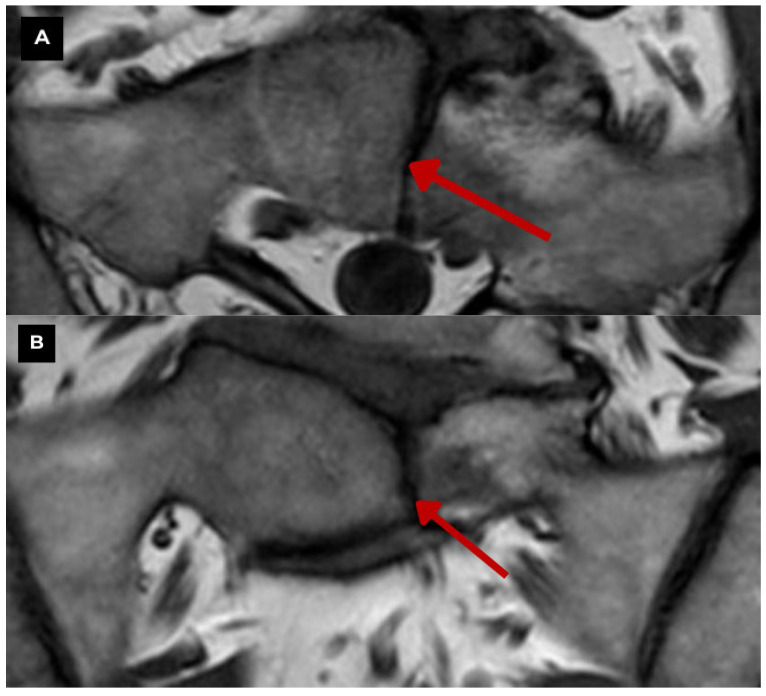

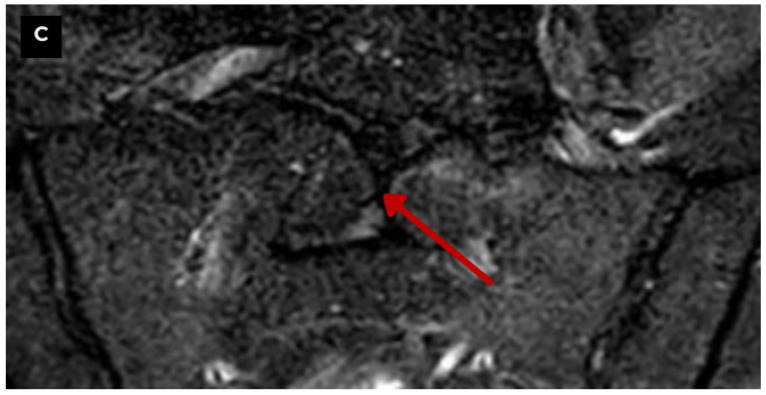
Second patient. Sacral MRI of a 39-year-old male. (**A**) T1-weighted sequence axial zoomed in. and (**B**) T1-weighted sequence TSE coronal zoomed in. Both show the midline sagittal cleft through the body of the S1 vertebra with preserved margins, and it is consistent with a benign vertebral anomaly. The defect measured at 2.9 mm. (**C**) STIR coronal zoomed in. Demonstrates focal high signal along the margins of the S1 midline cleft, highlighting surrounding bone marrow edema adjacent to it on the left hemivertebra, which characterizes Modic type I changes in the endplates at L5-S1 and is the likely cause of local pain. **Red arrow**—butterfly vertebra. Modic type I changes can evolve into Modic type II changes over time. In comparison, the MRI of the first patient did not reveal any signs of Modic-type endplate changes. This shows how a single vertebral anomaly can have different clinical implications. Literature provides information on the impact of this pathology on disc structures and facet joints, causing their degeneration and playing a role in the pathogenesis of chronic back pain [2,3]. Therefore, the finding of such a pathology shows the need for a detailed and multidisciplinary approach to diagnosis and treatment. This case highlights a relatively unusual vertebral pathology and underscores the necessity of a multidisciplinary approach.

## Data Availability

The original contributions presented in this study are included in this article; further inquiries can be directed to the corresponding author.
